# Conventional analysis of movement on non-flat surfaces like the plasma membrane makes Brownian motion appear anomalous

**DOI:** 10.1038/s42003-018-0240-2

**Published:** 2019-01-08

**Authors:** Jeremy Adler, Ida-Maria Sintorn, Robin Strand, Ingela Parmryd

**Affiliations:** 1 Science for Life Laboratory, Medical Cell Biology, Uppsala University, Uppsala University, Box 571, 751 21 Uppsala, Sweden; 2 Department of Information Technology, Uppsala University, Box 331, 751 05 Uppsala, Sweden; 3 Institute of Biomedicine, The Sahlgrenska Academy, University of Gothenburg, 405 30 Gothenburg, Sweden

## Abstract

Cells are neither flat nor smooth, which has serious implications for prevailing plasma membrane models and cellular processes like cell signalling, adhesion and molecular clustering. Using probability distributions from diffusion simulations, we demonstrate that 2D and 3D Euclidean distance measurements substantially underestimate diffusion on non-flat surfaces. Intuitively, the shortest within surface distance (SWSD), the geodesic distance, should reduce this problem. The SWSD is accurate for foldable surfaces but, although it outperforms 2D and 3D Euclidean measurements, it still underestimates movement on deformed surfaces. We demonstrate that the reason behind the underestimation is that topographical features themselves can produce both super- and subdiffusion, i.e. the appearance of anomalous diffusion. Differentiating between topography-induced and genuine anomalous diffusion requires characterising the surface by simulating Brownian motion on high-resolution cell surface images and a comparison with the experimental data.

## Abstract (web summary)

Jeremy Adler et al. simulate simple diffusion on non-flat, including cell, surfaces and introduce the shortest within surface distance. They show that ignoring topography understates the diffusion rate and can cause simple diffusion to appear as anomalous diffusion, a remedy is proposed.

## Introduction

There is a marked discrepancy between the measured diffusion of both lipids and proteins in artificial and biological membranes of about 5–20 times^1^. Explanations include hop diffusion^2^, transient anchorage^3^, molecular crowding^4^, fixed obstacles^5^ and membrane domains^6^, any of which could produce anomalous rather than Brownian diffusion. A linear relationship between the MSD (mean squared deviation) and time characterizes simple diffusion and a departure from this relationship indicates that additional factors influence the pattern of movement of the observed molecules or particles, i.e. that the diffusion is anomalous.

The prolonged and precise tracking of single particles on the surface of living cells by light microscopy is a technological triumph^7,8^ and analyses of the resulting single particle tracks (SPT) underpin contemporary models of the plasma membrane, e.g., hop diffusion^2,9^. Hop diffusion could be caused by a coherent network of barriers that compartmentalize the plasma membrane, with the diffusion within a compartment being relatively unconstrained while changing compartments is difficult^10^. This would give rise to relatively rapid short-term diffusion and reduced rates over longer timescales—a pattern observed on the plasma membrane. Stimulated emission depletion—fluorescence correlation spectroscopy (STED-FCS) also reports differences in the long-range and short-range diffusion coefficients but with a much lower confinement strength^11^, the discrepancy being explained by the larger experimental errors in SPT^12^.

A critical, though generally unstated, assumption in the analysis of SPT, and other methods for estimating diffusion in membranes like FCS and fluorescence recovery after photobleaching (FRAP), is that the surface is both locally flat and aligned with the imaging plane. Employing this assumption is common practice and it justifies the use of 2D tracking and distance measurements, but the widespread use of the assumption is somewhat remarkable since it was already acknowledged that topography could influence diffusion measurements in early FRAP studies^13,14^. However, other early FRAP studies did not find a role for topography, causing some confusion^15,16^. It has also been noted that membrane undulations can cause large errors in the interpretation of FCS data^17^. The movement of particles is, nonetheless, usually analysed using the 2D MSD of the shortest distances between sequential positions^18^.

The critical assumption that living biological membranes are locally flat lacks experimental support. A wide range of cell types examined live by hopping probe ion conductance microscopy, a non-contact surface scanning method, were shown to have ridges, undulations and projections and none were even locally flat^19^. This invalidates 2D interpretations of cell surface diffusion data, which systematically underreport the true rate of diffusion. For instance, a convoluted surface that halves the 2D measured distances would, as the calculated diffusion is based on the MSD, reduce the apparent diffusion by a factor of four. It therefore seems likely that the discrepancy between diffusion in the plasma membrane and in model membranes, that are reasonably flat, has been overestimated. Membrane topography also is of crucial importance for how we perceive cellular processes like cell signalling, cell adhesion and molecular clustering^20,21^.

There is a critical difference between measuring movement in a homogenous volume and on a homogenous surface; in a volume movement is unrestricted while a particle or molecule on a surface is confined. The MSD is the basis for SPT analysis. It disregards the path taken and uses the net movement measured by the straight-line-distance. The main advantage of the MSD is that it, unlike velocity, makes the measured motion independent of the frequency of observation; for Brownian motion, the MSD has linear relationship with time. Within a volume or on a flat surface the shortest distance is always a possible path, but on non-flat surfaces, the shortest route frequently departs from the surface, which is physically impossible for membrane components. The Euclidean (linear) distance between successive locations of a particle moving in a surface that is neither flat nor smooth therefore consistently understates the length of the shortest possible path.

Different approaches for simulating diffusion on uneven static and fluctuating biomembrane surfaces have been reported^22–25^. One approach uses level sets^26^ together with embedding the surface in a small annular region^24,25^. In another approach, random walk is used^22,23^. The simulation method in^22^ has been further developed to encompass moving surfaces and the effect of any particle-induced curvature^27^.

In this study, we simulate diffusion on surfaces with different topographies and compare commonly used measures of distance with a new measure. We show that the diffusion coefficient measured using the MSD varies substantially for different surface topographies and as a consequence for different regions of a cell surface. Moreover, we demonstrate how topography can create the appearance of anomalous diffusion.

## Results

**Fig. 1:**
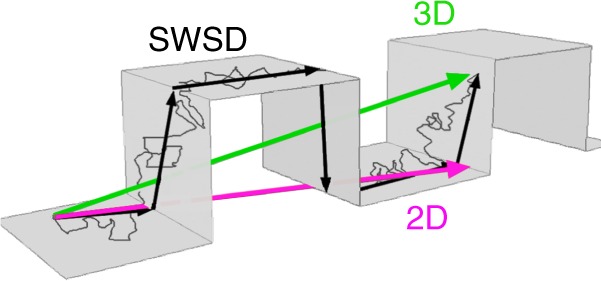
Different measures of a distance. Arrows mark the 2D, the 3D and the shortest within the surface distance between two points in a folded membrane. Note that both the 2D and the 3D distances require that the molecules leave the membrane

In diffusion studies of the plasma membrane, topography is frequently ignored and analyses that allow the diffusing species to leave the surface are commonplace (Fig. 1). To examine how topography affects diffusion patterns and the appearance of anomalous diffusion we have simulated diffusion on surfaces with different topographies, including cells, and evaluated commonly used measures of diffusion and introduce the shortest within surface distance (SWSD).

### Variations in the number of neighbours in simulations

First a way to represent a surface with topographical features as a grid is required. We used an orthogonal grid because this format is generated by most imaging systems. Like any grid, an orthogonal grid is only an approximation of a complex surface like the plasma membrane since the nodes are connected via 90° angles, whereas most biological surfaces are rounded. On a flat surface with no obstructions each node, except those at the edges, has the same number of neighbours, four in our simulations. The same holds for a surface with orthogonal folds; a folded surface that when unfolded is seen to arise from an intact sheet. We refer to these surfaces as folded and differentiate them from a second class of surfaces that we refer to as deformed; surfaces that can only be created from a flat sheet by differential stretching.

**Fig. 2:**
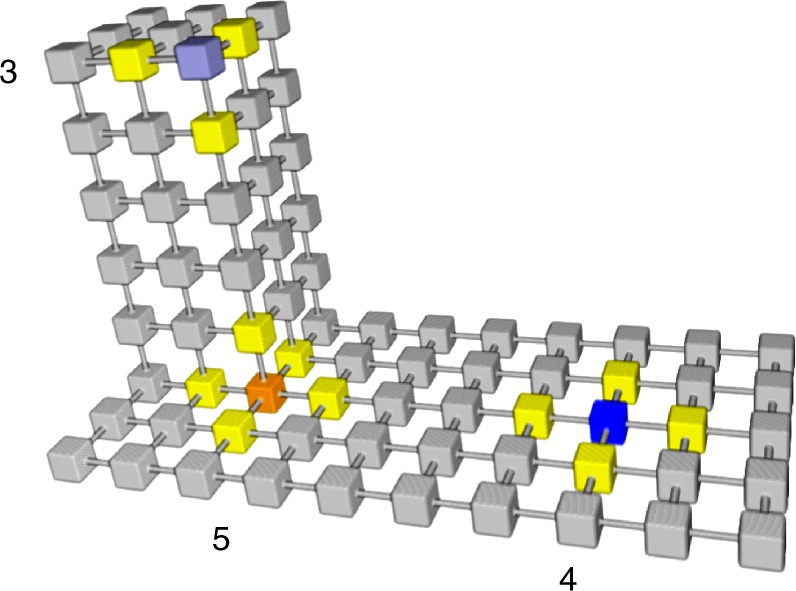
Surfaces comprise nodes with differing numbers of neighbours. Single nodes with three neighbours (blue/grey) are found at external corners. Nodes with four neighbours make up the bulk of the surface, a single example is shown in blue. Single nodes with five neighbours are found at internal corners (orange). The neighbouring nodes are all shown in yellow

On a folded surface every node has four neighbours while on deformed surfaces most nodes still have four neighbours, but there are also external and internal corners where nodes have three or five neighbours respectively (Fig. 2). The variation in the number of neighbouring nodes affects how the probability distribution subsequently spread, causing apparent anomalous diffusion as described later.

Simulated movement over a non-flat surface is more complicated than over a flat surface since there are six potential orthogonal moves of which between three and five remain within the surface (Fig. 2). To accommodate this, probability distributions were generated iteratively. In each cycle 50% of the particles in each node were redistributed among the neighbouring nodes that form the surface.

### Topography effects how particles spread

Movement can be displayed in a series of probability distribution maps, which show the likelihood of a single particle appearing at each position. This can be computed by iteratively simulating the diffusion of particles originating at a single position. The process is often expressed by differential equations. We used an all discrete approach, where no assumptions or approximations about the surface topography are required, interpreted the surfaces as graphs and applied the theory of random walks on graphs^28^.

Although not always acknowledged, it is well-known that there is substantial variability in the tracks of single particles even on flat homogenous surfaces^29^. This is illustrated by sixteen tracks (Supplementary Figure 1a). A consequence of the variability is that for some complete tracks or parts of single tracks the particles appear to undergo a range of non-Brownian diffusion.

The high variability of SPTs also means that many tracks are required to establish a representative MSD, illustrated by the difference between the distribution of endpoints for 16 and 64,000 tracks and the asymptotic behaviour calculated for spreading from a single starting point (Supplementary Figure 1b–d). To disentangle the contribution of the surface topography to the measured diffusion from genuine sources of anomalous diffusion we illustrate and investigate the surface topography contribution. We used a method based on probability propagation from a common starting position thus covering all possible movement and thus eliminating randomness.

**Fig. 3:**
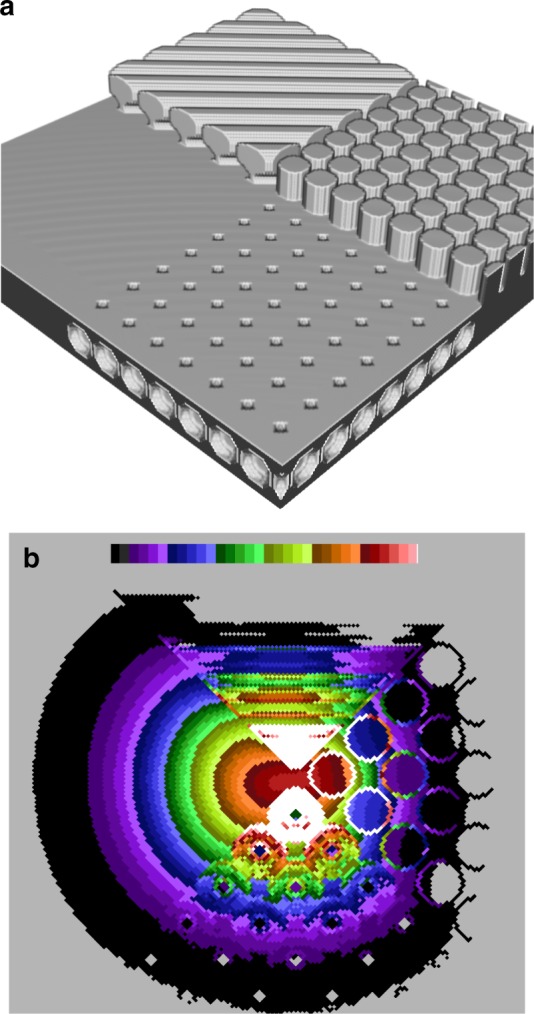
The topography of the surface affects the spread of particles. **a** Four different surfaces (flat, ridges, pillars and invaginations) and **b** the probability of finding a particle at any node after 1600 iterations. The starting position was in the centre where the four different surfaces meet. The probability distribution is displayed as a summed Z-projection contour plot, using a log-scale to display the wide range of probabilities

A surface with four different quadrants, one flat and horizontal and three resembling biological features, lamellipodia (ridges), filopodia (pillars) and invaginations (caveolae/endocytotic vesicles), was created (Fig. 3a). Diffusion from the central point was simulated for 1600 iterations and displayed by converting the volume into a 2D image using a summed Z projection (Fig. 3b). To illustrate the spread and wide range of probability a log-scale has been used.

On the horizontal and flat surface (left) the probability distribution has, as expected, a smooth concentric gradient (Fig. 3b), while on the surface with longitudinal folds (top), the gradient is not smooth, partly due to the summed projection display. That the probabilities fall with distance from the central starting point is most evident in the centre of the top quadrant; the pattern along the sides involves exchange with the two neighbouring quadrants. The pillars (right) have probabilities that are lower at their flat tops. In addition, there appears to be an accumulation of particles at the sides of the pillars, an artefact of the summed projection that in a 2D analysis could be misinterpreted as reduced or even non-movement, i.e., binding. On the surfaces with invaginations (bottom), the probability is lower at the centre of the invaginations, which could be misinterpreted as exclusion.

### The SWSD correctly measures diffusion on folded surfaces

**Fig. 4:**
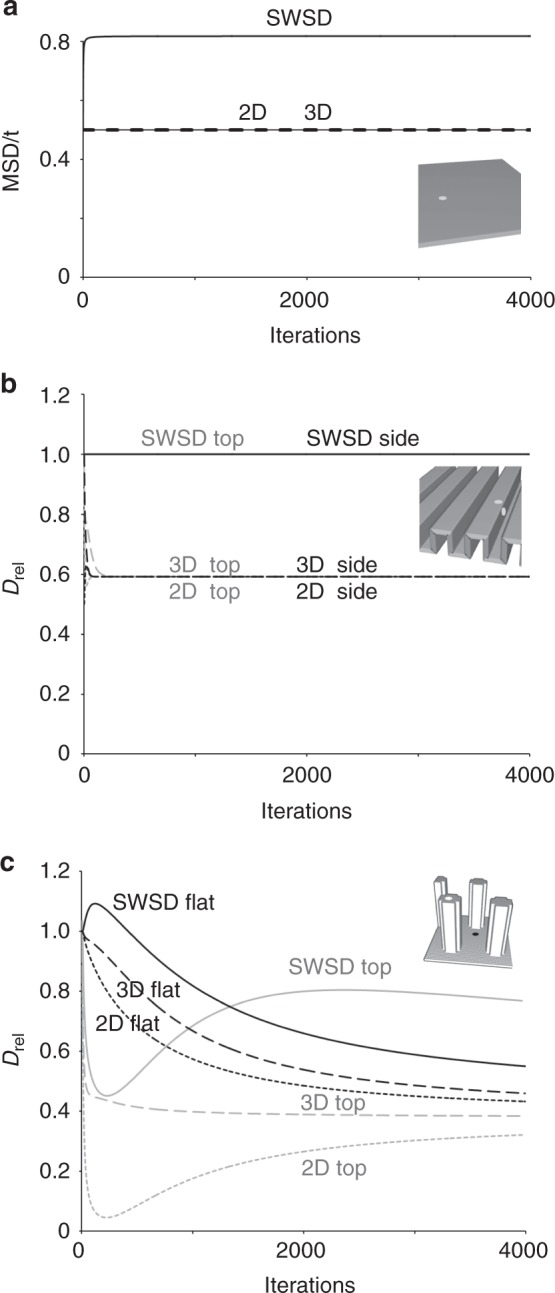
The shortest within surface distance accurately measures diffusion in folded, and outperforms current methods on deformed surfaces. **a** A flat and horizontal surface. **b** A surface with uniformly sized and spaced parallel ridges of four nodes height and four nodes width with two nodes spacing. **c** A surface with regularly spaced pillars (5 × 5 node base with the corners indented that rise 15 nodes above a horizontal surface on a hexagonal grid with 12 node spacing). Probability distribution simulations were launched at the positions indicated. Diffusion was measured using 2D Euclidean, 3D Euclidean and the shortest within surface distance, in **a** shown as the MSD/t and in **b** and **c** *D*_rel_ expressed relative to the measurements on a flat surface

To assess the impact of surface topography a flat surface was used to obtain baseline diffusion coefficients and calculating *D*_rel_, the rate relative to a flat surface (Fig. 4a). Note that the substantial difference between the Euclidean and SWSD MSD/t arises from differences in how distances are measured; the Euclidean distances are straight lines (the 5 on a 3,4,5 triangle). The SWSD was measured using an orthogonal propagation (the city block distance, 7 for a 3,4,5 triangle), making the distances measured with the SWSD on a flat surface longer. This difference is factored out by subsequently using *D*_rel_ (Fig. 4b). On a folded surface with ridges, the measurements from the 2D Euclidean distances (2D) and 3D Euclidean distances (3D) underestimated the diffusion compared with an unfolded horizontal surface (Fig. 4b). The location of the start position clearly affects the 2D measured diffusion. When starting on a horizontal part of the folded surface, the 2D measurement deviated from that on a flat surface (*D*_rel_ = 1) underestimating the diffusion after a few iterations once the diffusion front reaches the first fold, whereas starting at the centre of a vertical part of the surface resulted in a substantial and continuous underestimation of the diffusion. The 3D measurements are initially independent of the starting position and matched *D*_rel_ until the particles reach a corner where horizontal and vertical surfaces meet. Note, the 2D and 3D eventually converge at 0.59 that is independent of the starting point and reflects the fraction of vertical features. The convergence of 2D and 3D may at first seem surprising, but is a consequence of adding small topographical features to a horizontal surface. As the probability distributions spread the height component in the 3D distance relative to the total distance progressively drops leading to convergence, which would not occur if topographical features were on a deformed surface like a cell. When distances were measured using the SWSD, i.e., without leaving the surface, the measured diffusion matched that on the flat surface (Fig. 4b). The SWSD was accurate regardless of the relative orientation of the ridges, whereas the 3D reported a difference when the orientation of consecutive ridges changed (Supplementary Figure 2). For a surface with re-entrant features, the 2D and 3D were dependent on the starting position and again underestimated the diffusion while the SWSD was accurate (Supplementary Figure 3). In summary, the SWSD effectively unfolds surfaces, producing the correct distance and therefore correct diffusion measurements.

### The SWSD is not perfect on deformed surfaces

The plasma membrane of eukaryotic cells and membranes covering organelles is not well represented by a flat or a folded surface. We therefore ran simulations on surfaces with greater complexity, i.e., deformed surfaces with topographical features that cannot be obtained by folding a flat surface. First, we used surfaces with pillars, reminiscent of cellular protrusions like filopodia and cilia, with different starting positions (Fig. 4c). The pattern of diffusion was markedly different from that on a flat surface and varied with the starting position, reflecting both how quickly pillars, and for the first few iterations how many vertical neighbours and corners, were encountered. 2D were only close to *D*_rel_ when the starting point was in a horizontal part of the surface and then only briefly. Over the first few iterations, 3D match *D*_rel_ since the path does not leave the surface. The SWSD outperformed the 2D and 3D but importantly did not return the value expected for a flat surface. On a surface with small bumps laid out in a dense regular grid, i.e. a highly deformed surface, the SWSD still performed better than the 2D and 3D (Supplementary Figure 4).

**Fig. 5:**
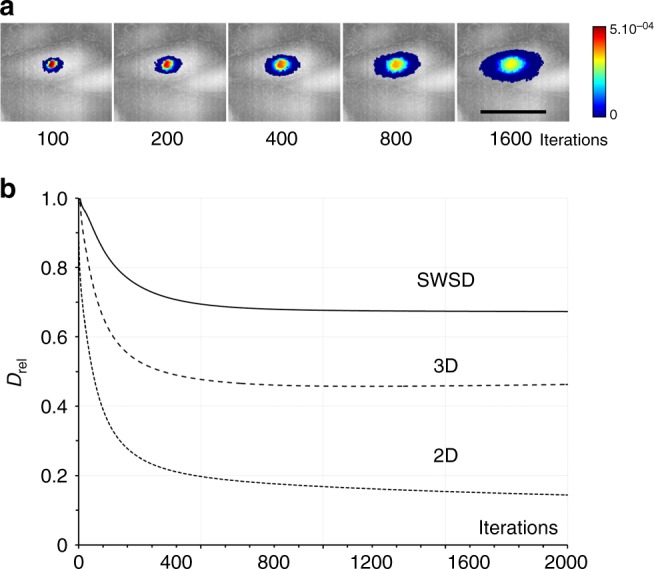
The SWSD greatly improves diffusion measurements on cell surfaces. A high-resolution topographical map of A6 epithelial cells from hopping ion conductance microscopy. A probability distribution simulation was launched close to the crown of a cell and iterated 2000 times. **a** The probability of finding a particle at any node after the indicated number of steps displayed as a summed Z-projection using a log-scale with low intensity made transparent to visualise the underlying cells is shown as a contour plot. **b** The diffusion expressed relative to a flat and horizontal surface (*D*_rel_). Scale bar 25 μm

A6 epithelial cells were used as a more physiological surface. Height-coded images were obtained by high-resolution hopping probe ion conductance microscopy^30^. The simulation was launched at the crown of a cell (Fig. 5a). Both 2D and 3D reported a substantial reduction in the apparent rate of diffusion (Fig. 5b), the 2D by more than a factor of five. Importantly, the 2D progressively dropped, i.e. did not reach a plateau. The SWSD outperformed the other methods and provided a better, but not a perfect match with measurements made on flat surfaces.

### Confined diffusion appear when topography is ignored

Hop diffusion, like other types of confined diffusion, is characterised by a fall in the rate of diffusion at longer timescales, appearing as a deviation from a straight line when the MSD is plotted against time. This is exactly what is seen when diffusion measurements from both folded, deformed and cell surfaces are made with 2D and even 3D (Supplementary Figure 5). Note that the characteristic deviation from a straight line vanished on the folded surface when the SWSD was used and was greatly reduced on the deformed and cell surfaces.

### Topography can cause both super- and subdiffusion

**Fig. 6:**
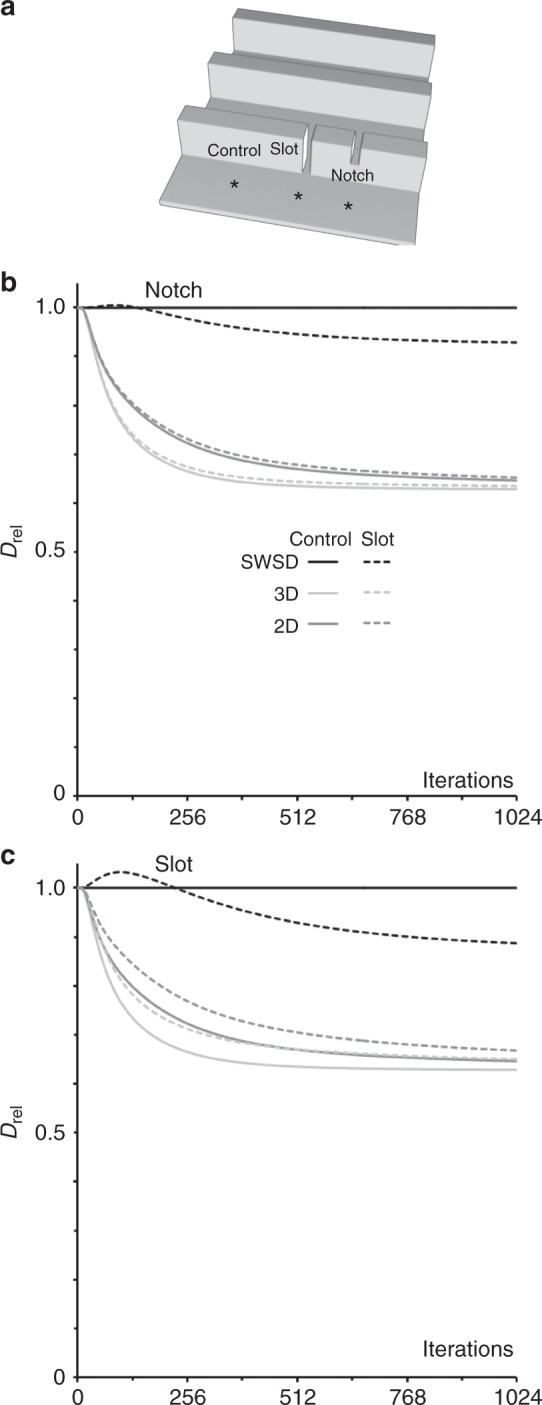
Gaps in a folded surface creates short-lived superdiffusion. **a** A surface with uniformly sized parallel ridges seven nodes high and three nodes wide at 12 node intervals in a 1023,1023,9 volume. Simulations run with a single defect in otherwise perfectly folded surfaces, either a one node wide slot or notch. The start point for the diffusion simulation was midway between the defect and the next ridge or for the control midway between two ridges, illustrated with asterixes. The diffusion coefficients for the ridges with a slot (**b**) and notch (**c**) measured using 2D, 3D and the SWSD expressed relative to a flat and horizontal surface (*D*_rel_)

**Fig. 7:**
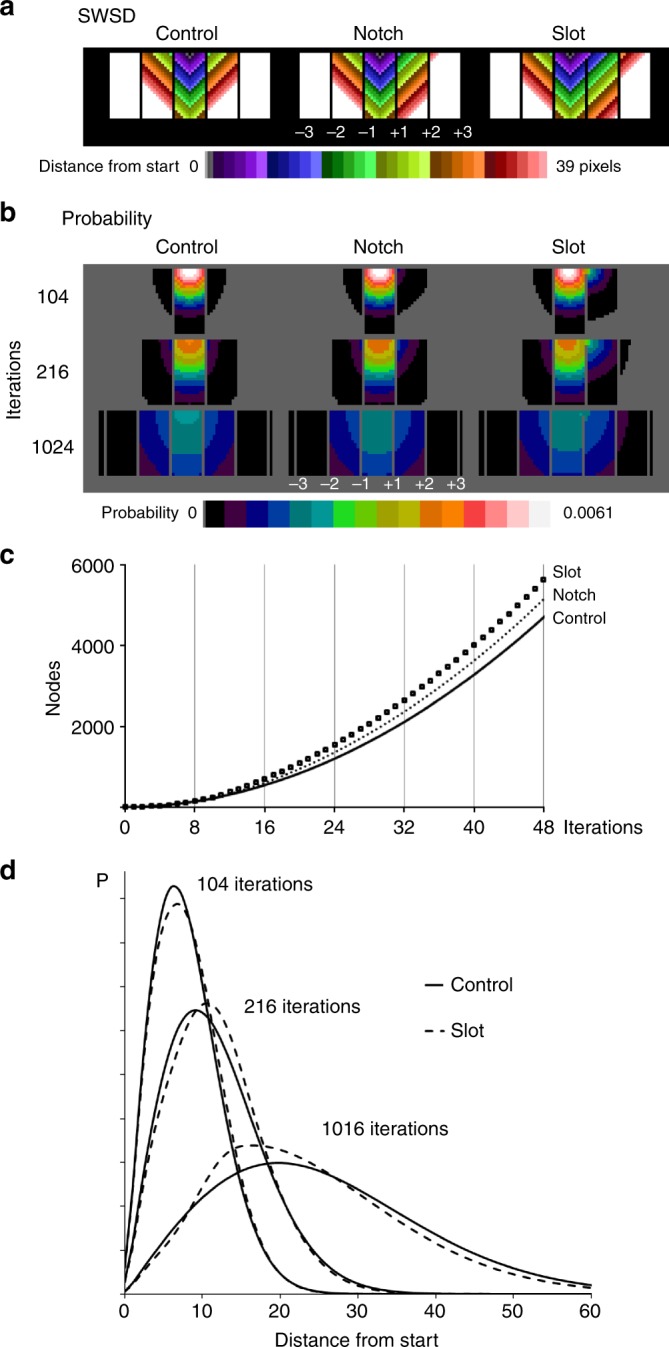
Topographical features can cause sub- and superdiffusion. Detailed examination of the SWSD diffusion measurement with the surfaces shown in Fig. 6. **a** Distances measured with the SWSD from the start point for the three surfaces. The stepped false colour look up table, which can also be described as a contour plot, covers the first 0–39 nodes and the ridges are indicated by vertical lines. The start point was midway between ridges −1 and +1 and at the top of the image, being the lowest intensity node in **a** and at the maximum probability in **b**, the centre of the white area. The defect is in the +1 ridge to the right of the start point. **b** Probability distributions at increasing numbers of iterations corresponding to the peak superdiffusion (104), the return to normal diffusion (216) and the maximum subdiffusion (1024) in Fig. 6. To include a wide range of probabilities the look up table uses the square root of the raw data. The ridges appear as vertical lines. **c** The number of nodes included in the spreading probability distribution increase with iterations. **d** Comparison of the probability for different distances from the start position at 104, 216 and 1024 iterations

Surprisingly *D*_rel_ values above one, i.e. superdiffusion, were observed with the SWSD over the initial iterations in several of our simulations (Figs. 4c and 6). Intuitively, superdiffusion in the absence of assistance like a motor protein to provide directionality to nominally freely diffusing molecules seemed unlikely and we investigated its origin. It transpires that superdiffusion can be a consequence of topography and arises when the diffusion front reaches more nodes than those encountered on a flat surface, e.g. when the front on a flat part of a surface encounters the bottom corner of a pillar. This is illustrated for a surface where a single slot or a notch has been inserted into a folded surface (Fig. 6). The increased area of spread originates at nodes at the corner of the base of the slot with five neighbours rather than the four neighbours found on flat parts of the surface (Fig. 2), since more neighbours allows the front to subsequently reach an increasing number of nodes and have a greater net movement (Fig. 7). The superdiffusion for the slot exceeds that for the notch simply because the five-neighbour nodes at the corners of the slot are closer to the starting point and provides access to more nodes earlier in the simulation. Analogously, the occurrence of nodes with three neighbours could cause subdiffusion manifested as initial dips in the *D*_rel_ plots when starting at the top of pillars (Fig. 4c). When the three neighbour external corner nodes are reached, the spread of the particles is subsequently reduced since the number of nodes to which the particles can spread is lower than it would be on a flat surface. However, as explained below, shortcuts can also cause subdiffusion.

A question is why SWSD superdiffusion is transient (Figs. 4c and 6c). The reason is a competition between the slot providing a shortcut that reduces the SWSD measurement to many nodes (Fig. 7a, b) and the slot providing access to more nodes which expands the probability distribution (Fig. 7c). Superdiffusion peaks at 104 iterations (Fig. 6c), when nodes beyond the slot are mostly occupied by molecules that passed through the slot (Fig. 7b). Compared with a surface with an intact ridge more particles have travelled further. After 216 iterations the *D*_rel_ versus iteration curve crosses 1, the expected value for Brownian diffusion (Fig. 6c). At the end of the simulation, much of the spread beyond the slot occurs using routes not utilizing the slot and the SWSD measurement, which shortcuts through the slot, therefore understates the distance travelled and the SWSD finally reports subdiffusion. This is manifested by a higher probability of finding particles for the surface of the slot at short distances (8–28 nodes) and a lower probability of finding them at longer distances (Fig. 7d). In summary, the existence of shortcuts creates brief superdiffusion and eventually the SWSD falls to a *D*_rel_-value below one, as seen with cells (Fig. 5).

## Discussion

Diffusion depends upon the properties of both the diffusing species and the medium, so a known medium can be used to characterise an unknown molecule or vice versa. On biological surfaces, the primary interest is to characterize the interaction of different molecules with a membrane and to establish whether movement is or is not Brownian where non-Brownian movement would require the recognition of a mechanism.

When the medium is a surface like the plasma membrane and 2D imaging is used the surface must be both flat and aligned with the imaging plane to correctly characterize the diffusing species in the medium—a requirement that is rarely acknowledged and, presumably, even less frequently met. Even then 2D images only permit 2D distance measurements. Misalignment of the surface with the imaging field or non-flat surfaces compromise the measurement of distances, since the commonly used 2D, but also 3D, measurements require the physically impossible, that diffusing molecules can leave and reenter the surface, i.e., illegitimate movement.

We set out to investigate whether it is possible to obtain diffusion coefficients that are independent of surface topography, which would permit the identification of genuine non-Brownian diffusion. In our quest, we revealed that in simulations emulating simple diffusion, topographical features alone can produce super- and subdiffusion. This is a critical finding when interpreting the movement of membrane molecules.

Vector calculus and partial differential equations are traditionally formulated in a continuous setting where the solutions are analytical expressions, often in a closed form. When computers are used to derive approximate solutions to the differential equations, discretizations of well-known continuous operators are usually applied. With this approach, it has to be established that the discretized, computed approximation and the desired analytical solution do not deviate too much, typically by assuring convergence with finer discretization. However, the approach we followed, discrete calculus, is inherently discrete and treats a discrete domain (essentially a graph) as its own entity without reference to an underlying continuum^31^. Consequently, since the concern in traditional discretization about convergence to a continuous solution is not a consideration in discrete calculus, we do not present a convergence analysis. Using our discrete approach also means that details on the cell surface not resolved by the imaging device are not represented in the subsequent topography analysis, which means that the genuine topography effects are likely to be larger than we report.

A critical observation is that 2D measurements always underestimate the net movement on non-flat surfaces, producing diffusion coefficients that are lower than those for a flat surface. On flattish areas movement would be only slightly reduced, which could be interpreted as a small reduction in the rate of diffusion, while where the movement has a substantial Z component, the dramatic fall in the apparent local rate diffusion risks being interpreted as binding or trapping^19^. Still 2D tracking of movement over the plasma membrane is common and variations in topography risk being mistaken for reduced diffusion and as indications of anomalous diffusion.

It follows that at least some of the apparent reduction in the rate of movement and the trapping observed in plasma membrane diffusion studies is attributable to 2D imaging, 2D distance measurements and topography^19^. In addition, the use of large gold particles could lead to erroneous results^12^ and the reduction in the long-term diffusion, i.e. the estimated difference between diffusion in model membranes and the plasma membrane, has been shown to be halved with the small lipid probes used in STED-FCS compared with gold-particle SPT-studies^11^. Interestingly, the lipids studied were found to undergo confined diffusion in an Arp 2/3-dependent, i.e. actin polymerisation-dependent, manner. The confined diffusion was mostly seen at the lamellipodia- (also Arp 2/3-dependent) rich cell edge, suggesting a contribution of cell topography to the residual anomalous diffusion.

Another phenomenon that can reduce diffusion and account for differences between model and cellular membranes is molecular crowding, i.e. that the protein to lipid ratio in biological membranes is relatively high, making large membrane areas inaccessible^4^. That crowding plays a role was supported by a recent study comparing diffusion in giant unilamellar vesicles, giant plasma membrane vesicles (GPMVs) and the plasma membrane of intact cells, where diffusion in the former (containing mostly lipids) was about five times faster than in the GPMVs and 10–25 times faster than in cells^32^. The difference between the cells and GPMVs is probably caused by the cells having a more variable topography. Interestingly, the measurements were performed on the basal part of the cells, where non-flat topographical features are considered to be less prominent than at the apical cell side. However, they still exists^33^.

In an isotropic medium, identical diffusion coefficients could be calculated from SPT measured in one, two or three dimensions. However, membranes are continuous surfaces and not isotropic. A related question is whether on deformed surfaces switching from 2D to 3D distance measurements is sufficient to produce topography-independent diffusion coefficients. We find that while 3D measurements are clearly a substantial improvement on 2D, especially over short timescales, they ultimately fail because the 3D does not recognize that movement is confined to the surface.

Interestingly, the reduced movement recorded with 3D in deformed surfaces falls with time. This is because the 3D ignores the topography and therefore is not confined to the surface. At longer times the difference between the shortest linear distance and the actual movement increases, as more paths include non-horizontal parts of the surface. The MS(quared)D gives extra weight to longer tracks, explaining why confined diffusion becomes more apparent at longer intervals.

On a flat surface straight-line distances are always possible, in the sense that a particle could take this route, while on a non-flat surface most 3D straight lines are impossible, because they leave the surface. This created our rationale for introducing the SWSD, i.e. distances must remain within the surface. The SWSD produces diffusion coefficients that are closer to those on a flat surface and less dependent on the topography than the 3D measurements. Importantly, in our simulations a deviation of the SWSD from one suggests anomalous diffusion, but is really only reflecting apparent anomalous diffusion, since it is caused by the topography and unrelated to the interactions between the diffusing species and the surface, i.e. the primary question in most studies. The deviation reflects the prevalence of folds and deformed topographical features of which the folds are accurately measured by the SWSD, but even the SWSD understates movement on the deformed parts of the surface.

Requiring measurements over a surface to remain in the surface is an improvement on 2D and 3D and the SWSD provides the best measure of movement in every simulation. On folded surfaces the SWSD is accurate, but on deformed surfaces, it still underestimates the actual movement. This arises because the MSD is based on the net movement and ignores the path, which on a deformed surface does not correctly characterize the actual movement. The SWSD finds the shortest route, analogously to taking a pass through a mountain range, which is representative of the efficient route chosen by hikers, but underreports the relatively directionless and longer routes taken by mountain goats. This makes the SWSD an imperfect proxy for the actual movement. When a shortcut is present, initially the calculated diffusion increases since the shortcut provides access to larger areas and increased spreading dominates. Later, the relatively small actual flux through the shortcut is overwhelmed by the majority of the diffusing species whose routes did not utilise the shortcut. Net movement is then underreported by the shortened SWSD, producing a fall in the measured diffusion. This caveat means that, even if the precise topography of a biological membrane is known and the SWSD used, molecular movement and simulations however analysed cannot correctly report the rate of diffusion or differentiate between topography-induced and other causes of anomalous diffusion.

Our results suggest that the decrease in diffusion with time that is generally interpreted as confined diffusion, e.g., hop diffusion, could be caused by topography as demonstrated with the 2D measurements on cells. It should be noted that it has been argued that the MSD versus Δt is not ideal for spotting anomalous diffusion and is also incapable of assessing fractions of multicomponent populations of tracks and therefore the cumulative probability distribution of the square displacements rather than individual tracks should be analysed^34,35^. However, regardless of the method used to assess anomalous diffusion, the effect of topography must not be ignored, if the aim is to assess the interaction of molecules with the surface rather than characterising their spread over an unknown topography.

The SWSD provides the most accurate diffusion coefficients but still underestimates and distorts the pattern of movement. The ultimate solution lies in simulating simple diffusion over the surface to show the pattern that Brownian motion alone would produce and then comparing this with the experimentally observed pattern from SPTs or FRAP. It should then be possible to establish whether the experimentally observed non-Brownian movement exceeds that caused by topography. The major practical problem is how to obtain sufficiently detailed maps of the surface of living cells. In their absence, SPT and other techniques where diffusion is assessed, FCS and FRAP, should be interpreted with caution and interpretations involving topography considered before more elaborate models are invoked.

In our simulations, the topography of the surface per se does not alter the interaction between the particle and surface and therefore the mechanism underlying movement is identical on flat and non-flat surfaces. There are, however, scenarios where topography could directly affect diffusion for instance by blocking the entry of large and/or inflexible particles from areas of high curvature, as reported for proteins and thin membrane tubes^36,37^. Particles excluded from topographical features could diffuse more rapidly, which may explain the differential diffusion behaviour of synthetic lipids observed in cells at super lateral resolution^38^ and the remarkable fast diffusion of a cholesterol analogue^39^. It has also been reported that diffusion along the longitudinal direction in membrane nanotubes slows as the radius of the tube is reduced^40,41^, but note that this involves curvatures tighter than those found in cell membranes and the effect of the diffusion may be caused by crowding or changes in membrane tension and lipid packing that also restrict diffusion^42,43^. However, in cases where topography does effect the diffusion our protocol would identify the diffusion as anomalous. The exception being when the surface covers a thin tube, that although it is a foldable surface the SWSD would, like in the case with the shortcut, underestimate the actual distance moved around the axis of the tube with a full circle being measured as no net movement. A remedy could be to consider only the longitudinal movement which, at least when using the MSD versus Δt approach, would show Brownian movement^37^.

**Fig. 8:**
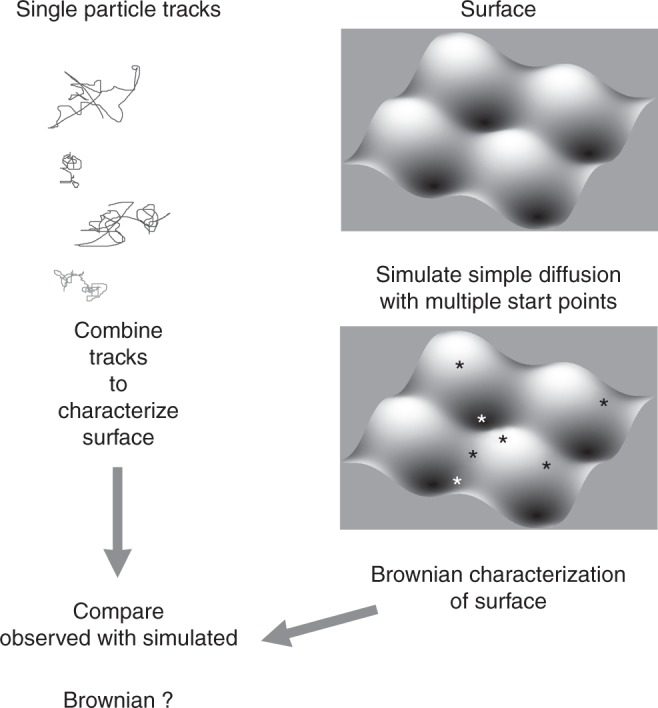
Scheme for disentangling the contribution of topography from other sources of anomalous diffusion

To determine the contribution of topography to any deviation of the diffusion from Brownian, the topography must be established, which is non-trivial given the dynamics, folding potential and thickness (<5 nm) of biological membranes. Our findings suggest a scheme for disentangling the topographical component from other sources of anomalous diffusion (Fig. 8). Firstly, create a 3D model of the surface. Secondly, run simulations with multiple start points covering the area of the SPT tracks producing probability distributions. Finally, assess whether or not the recorded tracks are consistent with the distribution generated in the simulations^44^. Note that, as visualised in Suppl. Figure 1, the variation between tracks is substantial, meaning that many tracks over the same surface are required to determine whether anomalous diffusion is indeed taking place^29^. That the start position is important for the simulations further emphasises the need for a large number of starting points for proper surface characterisation.

When topography has not been considered as a cause of anomalous diffusion, the norm, the resulting plasma membrane models are questionable since the primary objective is to understand the interaction of molecules with a biological surface, not to characterise spreading patterns over an unknown and varying topography. Establishing and factoring out topography needs to be done before invoking more complex explanations for reduced and anomalous diffusion – Occam rules.

In conclusion membrane topography has been identified as a cause of both the consistent underestimation of diffusion and the overestimation of apparent anomalous diffusion. Measurements are more accurate when the topography is known and distance measurements are kept within the surface, i.e., using the SWSD on a high-resolution 3D surface. Membrane topography itself can also cause apparent anomalous diffusion by allowing increased or decreased spread of the diffusion species. To factor out the topographically induced anomalous diffusion simulations of simple diffusion over the surface is required.

## Methods

### Creation of 3D surfaces

Non-flat continuous surfaces were made from an array of 6-connected cubic nodes within a 3D volume. The nodes that formed a continuous surface were usually connected to four of the six possible neighbouring nodes but nodes with three and five neighbours also featured (Fig. 2). Surfaces with longitudinal folds were created with parallel ridges of constant widths and spacing. Images with pillars and caveolae were created by repeatedly inserting a 3D structure pillar/caveola. Height-coded 2D images of cells were converted into surfaces within a 3D volume by placing the nodes at the *X*, *Y* & *Z* positions held in the 2D image and creating connections between them by adding vertical lines of nodes, producing a continuous surface.

### Generating single particle tracks

On flat surfaces tracks were made from sequential random moves to one of the 4-face connected nodes, determined by a sequence of random integers where each iteration (equivalent to time step) also included a 50% probability of moving.

### Diffusion simulation

Diffusion on surfaces was simulated using a sequence of random movements between adjacent nodes in a 2D array. The continuous diffusion equation is

1 $$\frac{{\partial u}}{{\partial t}} = \alpha \nabla ^2u$$

where *u* is concentration, *t* is time and *α* is the diffusion constant. In discrete calculus, the diffusion equation can be written as

2 $$\frac{{\partial u}}{{\partial t}} = - \alpha Lu$$

where *L* is the discrete Laplacian matrix operator here defined as

3 $$L\left( {i,j} \right) = \left\{ {\begin{array}{*{20}{l}} {\# {\mathrm{nodes}}\, {\mathrm{adjacent}}\, {\mathrm{to}}\, i} \hfill & {\mathrm{if}}\, i = j \hfill \\ { - 1} \hfill & {\mathrm{if}}\,i\, {\mathrm{and}}\, j\,{\mathrm{are}}\, {\mathrm{adjacent}} \hfill \\ 0 \hfill & {\mathrm{else}} \hfill \end{array}} \right.$$

where the value at *L* (*i*, *j*) is the number of nodes adjacent to *i* if *i* = *j* and −1 if *i* and *j* are adjacent^28^. Note that this is not a differential equation discretized in space, but a discrete differential equation, and that diffusion in discrete calculus when applied to graphs equals diffusion on graphs^45^. The value of *α* in the discrete diffusion equation determines what the diffusion should be along each edge in the graph, i.e. to each connected node corresponding to each neighbouring node.

To simulate the diffusion process, a lazy random walk^46^, with a 50% probability of moving was implemented iteratively. Hence, the probability of a move from a given node to any of its adjacent nodes depends on the number of adjacent nodes (neighbours), given by Eq. (5). For the most common case (four neighbours), the probability of a move to a particular neighbour is hence 0.125. The lazy random walk process is simply

4 $$u^{[t + 1]} = Du^{[t]}$$

where the matrix *D* is

5 $$D\left( {i,j} \right) = \left\{ {\begin{array}{*{20}{l}} {0.5} \hfill & {if\,i = j} \hfill \\ {\frac{{0.5}}{{\# {\mathrm{nodes}}\,{\mathrm{adjacent}}\,{\mathrm{to}}\,i}}} \hfill & {{\mathrm{if}}\,i\,{\mathrm{and}}\,j\,{\mathrm{are}}\,{\mathrm{adjacent}}} \hfill \\ 0 \hfill & {\mathrm{{else.}}} \hfill \end{array}} \right.$$

With this formulation, the probability of no move is 0.5 and the probability of moving to an adjacent node is 0.5. Thus, the probability of a move to a particular adjacent node depends on the number of neighbours.

In an empty (zero intensity) 3D floating-point array the start node on the surface was given a sufficiently high-intensity value representing the location of 100% of the particles. A 3D array that defined the surface was used to determine to which nodes movement was allowed. The resulting sequence of images shows the probability distribution from a single node. Distances from the origin were calculated as described below, using a geodesic distance transform. At regular intervals during the simulations the sum of the intensity at each distance from the start point was recorded.

### Analysis of diffusion

Each node in the image is represented by a node in a graph and the adjacency of each node is represented by a column in *D*. The analysis is initialized by setting one or more elements in a vector *v*_0_ to one, and the other elements to zero. In each iteration, the diffusion values are propagated by *v*_*t*_ = *Dv*_*t*−1_ generating a time course. The state at time *t* = *T*, *v*_*T*_, *v*_0 _is thus *v*_*T*_ = *D*^*T*^*v*_0_. To find the state after *m* iterations on an image of size *n*^2^, the matrix *D* with size in the order of *n*^4 ^should be multiplied with a vector *m* times. For large images like the ones used in this study, this is computationally very demanding. Therefore the size of the matrix *D* was limited by only considering a region around the single start point. The size of the region was increased as the diffusion-propagation approached the border of the region.

Measures of the distance between two-time points, *x*_1_, *y*_1_, *z*_1_ to *x*_2_, *y*_2_, *z*_2_ were performed using either:

- The Euclidean distance within a plane, which measures a straight line
  6 $${\mathrm{2D}} = \sqrt {\left( {x_2 - x_1} \right)^2 + \left( {y_2 - y_1} \right)^2}$$
- The Euclidean distance within a volume, which measures a straight line
  7 $${\mathrm{3D}} = \sqrt {\left( {x_2 - x_1} \right)^2 + \left( {y_2 - y_1} \right)^2 + \left( {z_2 - z_1} \right)^2}$$
- The shortest within surface distance (SWSD), is the shortest 3D distance that stays within the surface, i.e. the geodesic distance. The Euclidean distances were propagated to local neighbours within the surface. The complete geodesic distance transform was computed by a wave-front propagation approach^47^.

Diffusion coefficients were obtained from the relationship between the MSD and time by fitting a least square tangent to the MSD and expressed relative to movement on a flat surface (*D*_rel_). After each iteration the population MSD was calculated from the summed probability multiplied by the relevant distance squared.

### Software

Simulations and computations were performed in Matlab (The MathWorks Inc,. Natick, MA) and ImageJ^48^. Graphs were generated using Excel and figures were created using Adobe Photoshop. 3D rendered images were generated using 3D Viewer in ImageJ^49^ and 3D Slicer^50^.

### Code availability

The code is available in the folder of Supplementary Software 1.

## Supplementary information

Supplementary Information

Supplementary Software 1

Description of Additional Supplementary Files

Supplementary Data 1

Supplementary Data 2

Supplementary Data 3

Supplementary Data 4

## Data Availability

All data generated or analysed during this study can be recreated from the code supplied in the folder Supplementary Software 1. The code includes an example on how to generate a surface. The source files for the graphs in Figs. 4–7 are available as a Supplementry Data 1–4.
