# Supplementary material for: Conventional analysis of movement on non-flat surfaces like the plasma membrane makes Brownian motion appear anomalous
Source: Commun Biol. 2019 Jan 8;2:12. doi: 10.1038/s42003-018-0240-2 (PMC6325064; doi:10.1038/s42003-018-0240-2)
Supplement: Supplementary file 1 — Supplementary Information [file 42003_2018_240_MOESM1_ESM.pdf]

Supplementary figures

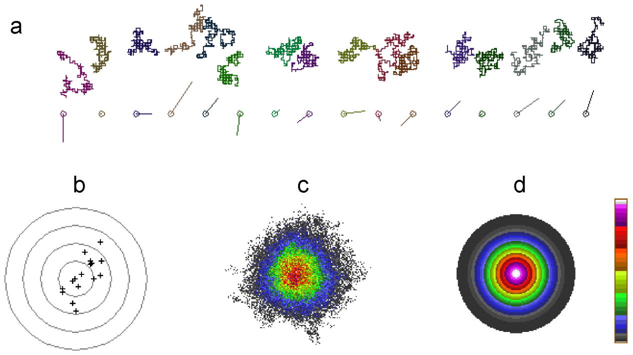

Supplementary Figure 1 The variability of tracks showing Brownian motion. (a) 16 randomly selected tracks with 1024 iterations with the distance between the starting and the end position shown below each track. (b) Distributions of 16, (c) 64 000 and (d) asymptotic behaviour of particles undergoing Brownian motion with (c) and (d) displayed as contour plots.

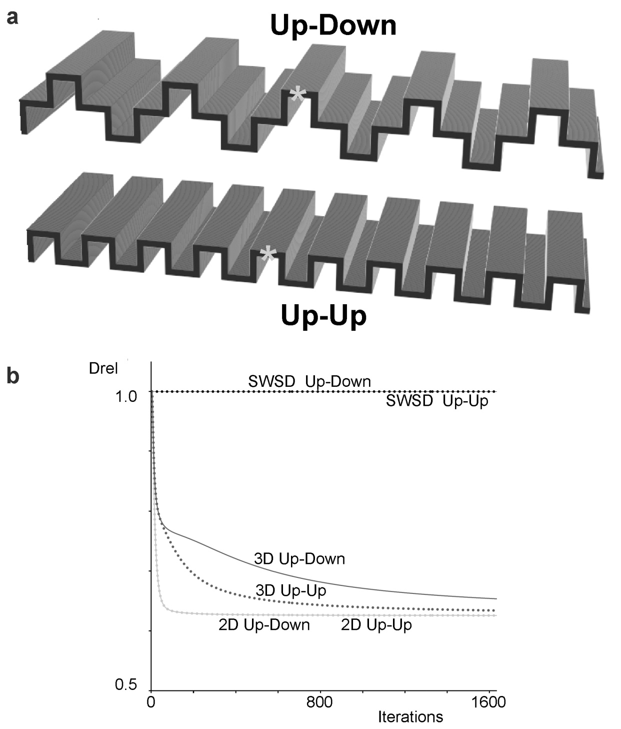

Supplementary Figure 2 The SWSD accurately measures diffusion in folded surfaces with folds regardless of direction. (a) Two folded surfaces with the same surface area but a different folding pattern were constructed. Each repeat of the folded surface fits into a 14 node wide and 11 node high cross section. (b) The diffusion coefficients in c measured using 2D, 3D and the SWSD expressed relative to a flat and horizontal surface (D_{rel}).

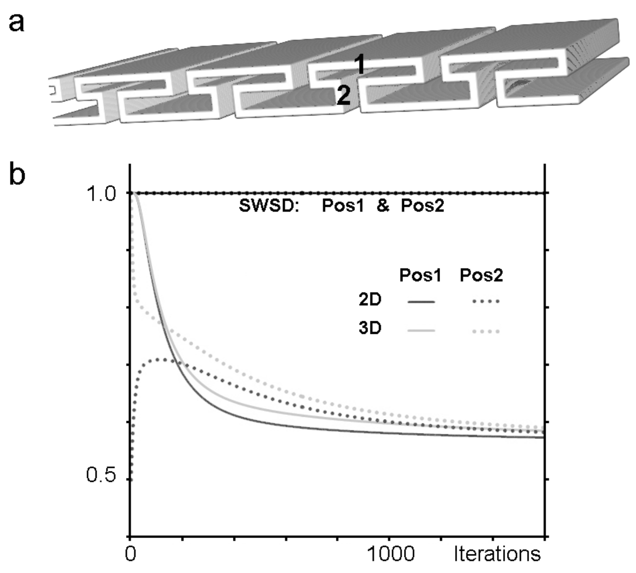

Supplementary Figure 3 The SWSD accurately measures diffusion in a folded surface with re-entrant features. (a) The start positions of the simulations are marked (*). Probability distribution simulations were launched at the positions indicated. (b) (c) The diffusion coefficients measured using 2D, 3D and the SWSD expressed relative to a flat and horizontal surface (D_{rel}).

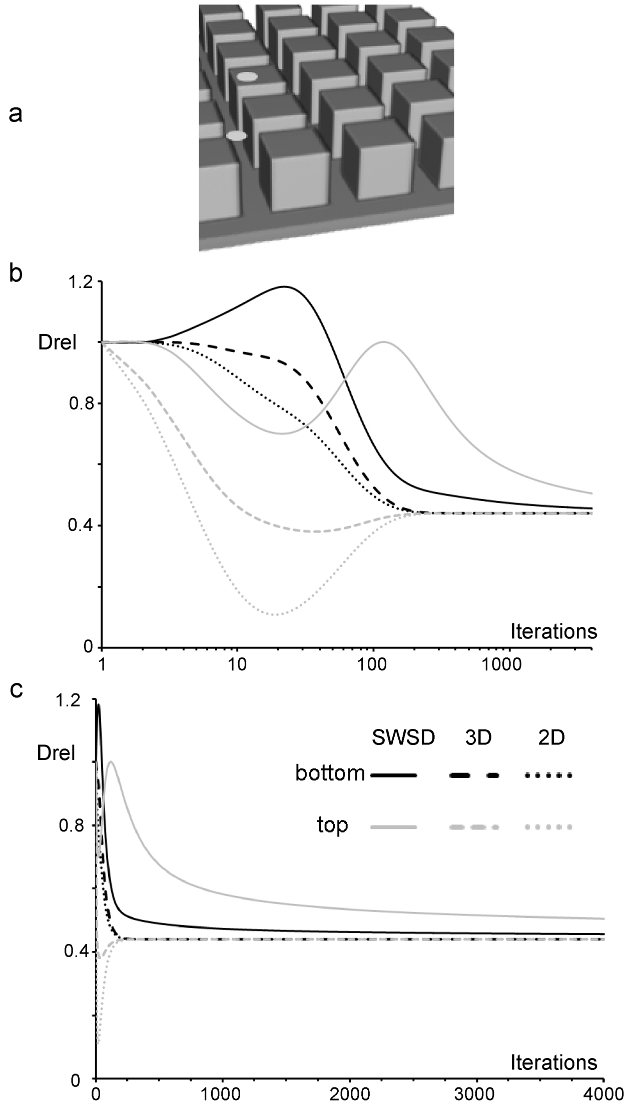

Supplementary Figure 4 The SWSD fails on highly deformed surfaces. (a) A surface with regularly spaced pillars, four nodes high and four nodes wide with two nodes spacing. Probability distribution simulations were launched at the positions indicated. (b, c) The diffusion coefficients measured using 2D, 3D and the SWSD expressed relative to a flat and horizontal surface (D_{rel}). The simulations comprised 4000 iterations. To visualise what is happening during the early iterations, a log-scale is used in b.

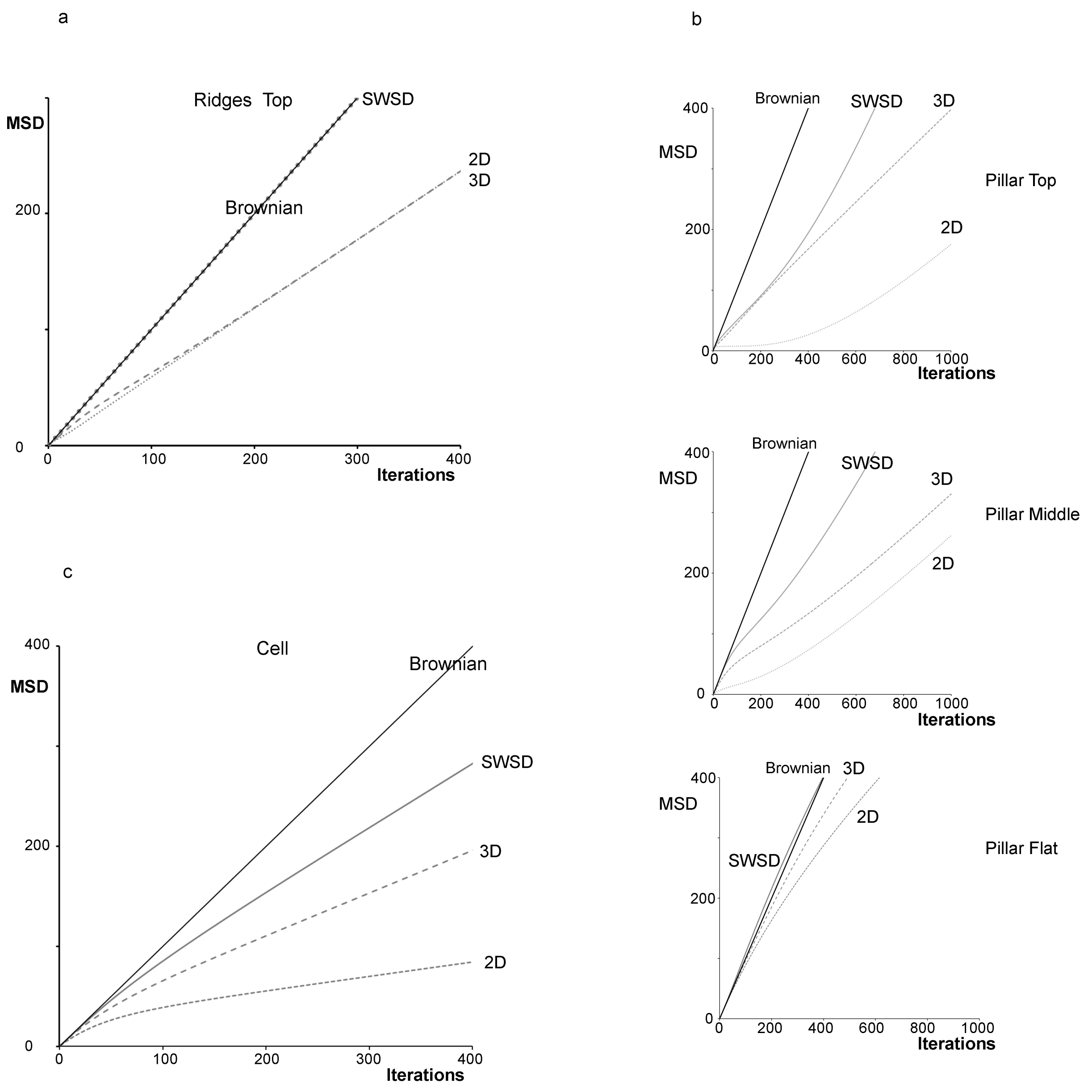

Supplementary Figure 5 Apparent anomalous diffusion can be the result of inappropriate analysis of diffusion data. Probability distribution simulations were performed and the MSD versus time for diffusion for 2D, 3D and shortest within surface measurements determined for (a) a folded surface with ridges (Fig. 4b), (b) a deformed surface with pillars (Fig. 4c) and (c) the surface of an A6 cell (Fig. 5) using the crown of the cell as the starting position. The area with the first 200 iterations has been enlarged to emphasise differences between the measurements. A deviation from a straight line at longer times is usually interpreted as confined diffusion.
