# Supplementary material for: Conventional analysis of movement on non-flat surfaces like the plasma membrane makes Brownian motion appear anomalous
Source: Commun Biol. 2019 Jan 8;2:12. doi: 10.1038/s42003-018-0240-2 (PMC6325064; doi:10.1038/s42003-018-0240-2)
Supplement: Supplementary file 3 — Description of Additional Supplementary Files [file 42003_2018_240_MOESM3_ESM.pdf]

<u>Description of additional supplementary items</u>

Supplementary Software 1 (zipped folder)
Compute_flat_diff.m - Computes diffusion coefficients for a flat surface.

RWEuclideanOct2018 - Computes diffusion coefficients on a surface of volume A by random walk from the start point.

CellDiffusion_RandomWalkExample - An example script for how to run cell diffusion simulations based on random walk.

disttrans.mexw64 - Binary matlab mex-file compiled for 64 bit Windows.

Supplementary Data 1
Figure 4 source data. Excel data sheet for the graphs in panels a-c, Figure 4

Supplementary Data 2
Figure 5 source data. Excel data sheet for the graph in Figure 5

Supplementary Data 3
Figure 6 source data. Excel data sheet for the graphs in panels b-c, Figure 6

Supplementary Data 4
Figure 7 source data. Excel data sheet for the graphs in panels c-d, Figure 7
